# The Role of *Klebsiella* in Crohn's Disease with a Potential for the Use of Antimicrobial Measures

**DOI:** 10.1155/2013/610393

**Published:** 2013-10-10

**Authors:** Taha Rashid, Alan Ebringer, Clyde Wilson

**Affiliations:** ^1^Analytical Sciences Group, King's College, 150 Stamford Street, London SE1 9NH, UK; ^2^Departments of Microbiology and Pathology, King Edward VII Memorial Hospital, Hamilton, Bermuda

## Abstract

There is a general consensus that Crohn's disease (CD) develops as the result of immune-mediated tissue damage triggered by infections with intestinal microbial agents. Based on the results of existing microbiological, molecular, and immunological studies, *Klebsiella* microbe seems to have a key role in the initiation and perpetuation of the pathological damage involving the gut and joint tissues in patients with CD. Six different gastroenterology centres in the UK have reported elevated levels of antibodies to *Klebsiella* in CD patients. There is a relationship between high intake of starch-containing diet, enhanced growth of gut microbes, and the production of pullulanases by *Klebsiella*. It is proposed that eradication of these microbes by the use of antibiotics and low starch diet, in addition to the currently used treatment, could help in alleviating or halting the disease process in CD.

## 1. Introduction

Crohn's disease (CD) is a chronic, progressive, and potentially disabling disease, characterised by relapsing and remitting episodes of transmural inflammation of the gastrointestinal tract which might be associated with arthritic manifestations [1]. Both CD and ulcerative colitis (UC) can be categorized under the name of inflammatory bowel disease (IBD). The differentiation between UC and CD is mainly based on clinical manifestations and the scale of bowel involvements. Patients with either CD or UC, however, are likely to have associated extraintestinal manifestations [2].

IBD is classified as one of the constituents of a group of diseases collectively termed spondyloarthropathies (SpAs). The other entities of this group include ankylosing spondylitis (AS), reactive arthritis, psoriatic arthropathy, and undifferentiated SpA [3]. There are certain features frequently associated with this group of diseases and these include spinal/sacroiliac arthritis, oligoarthritis, enthesitis, uveitis, negativity for rheumatoid factors, and a positive family history. 

A positive family history and a high degree of association of SpAs with HLA-B27 genetic markers have placed these conditions under the umbrella of what is called “B27 diseases” [4]. Furthermore, patients with CD seem to share more genetic, clinical, laboratory, immunological, and pathological features with AS patients [5–7].

CD is a relatively common condition involving millions of people all around the world. It usually affects a younger age group with a worldwide distribution. It has a high impact on the psychological condition [8], as well as the social status and work abilities [9] in patients with this disease. Based on these negative effects on the general health and welfare of patients with CD and the drawbacks of the currently used medical treatments as well as the increased likelihood of surgical complications in these patients, a search for the causative factor(s) together with a proposal for the use of an alternative therapeutic strategy involving eradication of the causative factors could be of crucial importance in the management of this disease.

## 2. Aetiopathogenesis

The incidence of CD has been increasing substantially in the last few decades, where more than 40,000 new cases in Europe and North America have been estimated to be diagnosed each year [10]. In a more recent analytical review study, it was shown that the incidence and prevalence of CD are increasing with time and in different regions around the world, particularly among the Caucasians [11]. The same increase in the incidence of CD was also noticed during the last few decades among the patients from East Asia [12]. These rises in the occurrence of CD could largely be explained by altered lifestyles and environmental exposures and/or changes in the dietary habits among the patients with this disease.

At least three interacting elements including genetic factors, intestinal microbes, and immune-mediated inflammation are involved in the pathogenesis of CD [13]. The most popular theory, however, is that the disease usually results from an abnormal immune response to certain enterobacterial pathogens present in the gut lumen.

Evidence from twin and familial aggregation [14] as well as genome-wide association [15] studies indicates that genetic factors play an important role in the aetiopathogenesis of CD (as shown in the lists below). Although many of the non-HLA genes such as NOD2/CARD15, IL23R, and CTLA4 [16] have been suggested to play a role in the susceptibility to develop CD until now, however, scientists have not been able to discover a gene which might solely contribute to the aetiology of this disease.

*Aetiopathogenetic Evidence for the Roles of Genetic/Microbial (Especially Klebsiella) Factors in CD*

*Evidence of the Genetic Involvement*

- Positive family history.
- A considerable increase in the risk of developing the disease among monozygotic twin of patients with CD.
- Higher risk of developing the disease among relatives of patients with CD or other entities of SpAs.
- Presence of HLA-B27 genetic markers in more than 50% of patients with CD having spondylitis.
- Significant association with NOD2/CARD15 and some other genetic mutations.
- Transgenic rats carrying human HLA-B27 genes are more prone to develop spontaneous bowel lesions.

*Evidence of the Environmental/Microbial Factors*

- More than 80% of dizygotic twins of patients with CD fail to develop the disease.
- Increased incidence of CD among friends and clustering of unrelated people as well as immigrants moving from low to high risk areas.
- Associated bouts of remissions and exacerbations with spatial and temporal variations in the incidence and the clinical course of the disease in CD.
- Increased gut mucosal leukocyte trafficking and enhanced microbial innate immune responses, as well as increased risk of developing the disease after appendectomy in patients with CD.

*Evidence of the Role of Klebsiella and Crossreactive Self Antigens*

- Increased load of *Klebsiella* culture from fecal matter in the large bowel.
- Occurrence of intercurrent *Klebsiella* intestinal colitis in patients with CD.
- Significantly enhanced antibody responses to *Klebsiella* microbes were found on many occasions in patients with CD when compared to healthy subjects.
- Increased antibody levels against collagens I, III, and IV, all crossreactive with *Klebsiella* pullulanase enzymes, were found in patients with CD when compared to healthy controls.
- Molecular similarities were found between different antigens present in *Klebsiella* nitrogenase and pullulanase “PulD” enzymes and HLA-B27 self-antigens. Furthermore, *Klebsiella* pullulanase “PulA” enzymes were found to possess a molecular structure similar to the trihelical shape of collagen I, III, and IV fibres.

The link between HLA-B27 and CD on the other hand is relatively more obvious especially in patients with associated spondylitis. For example, more than forty percent of patients with CD, especially those having spondylitis/sacroiliitis, were found to be positive for the HLA-B27 markers, but the prevalence of this gene in patients with CD without spondylitis/sacroiliitis merely parallels its frequency in the Caucasian general population [17]. Furthermore, HLA-B27 positive Caucasians are 20 times more likely to develop one or other entities of the SpAs, including IBD [18]. There is also evidence of high risk and cross-risk ratios among the first-, second-, or third-degree relatives to develop IBD or AS [19]. Moreover, in an experimental study by a group from Dallas, transgenic rats with high level of human HLA-B27 expression were found to be more prone to develop bowel inflammation with arthritis, and the severity of intestinal features was correlating with concentration of the large bowel microbial flora [20]. 

### 2.1. Evidence of the Role of Environmental/Microbial Factors in CD

Extensive evidence supports the role of certain environmental and/or microbial factors in the aetiopathogenesis of IBD and particularly in the causation of CD (as shown in the first three lists).

- A negative family history in more than 95% [21] and a low concordance rate among twins [14] of patients with CD support the role of environmental factors in the development of this disease.
- There is a report for an increased clustering of CD among unrelated individuals [22] and closely living friends [23], as well as amid second-generation immigrants moving from low to high risk areas [24]. In a previous study on a large Moroccan family and one parent with CD emigrated to Belgium, 4 out of the 8 children were found to have subsequently developed the disease [25]. Failure to find significant genetic variants with high incidence of CD among this family suggests the direct role of a major environmental factor which probably has been encountered in the new residing area and is not related to the sanitation in childhood.
- Patients with CD show intermittent variations in the clinical onset and activity during the disease course [26] as well as during different seasons of the year with associated concurrent intestinal bacterial infections [27].
- Increased incidence of CD after appendectomy [28], together with involvement of neutrophils and lymphocytes [29] in the gut inflammation as well as enhanced general immune response to enterobacterial antigens in these patients [30], supports the role of gut microbes in the aetiopathogenesis of this disease.
- Transgenic rats do not develop colitis under germ-free conditions, but when these rats are transferred into conventional environments, they develop signs of intestinal inflammation and colitis [31]. These results indicate that colonic microflora plays a key role in the development or exacerbation [32] of the intestinal inflammation resembling that occurring in patients with CD.

During the last few decades, various microbial agents [33], including also *Klebsiella pneumonia* [34], have been implicated to have a role in the aetiopathogenesis of CD. 

### 2.2. Evidence of the Role of *Klebsiella* in CD

**Figure 1:**
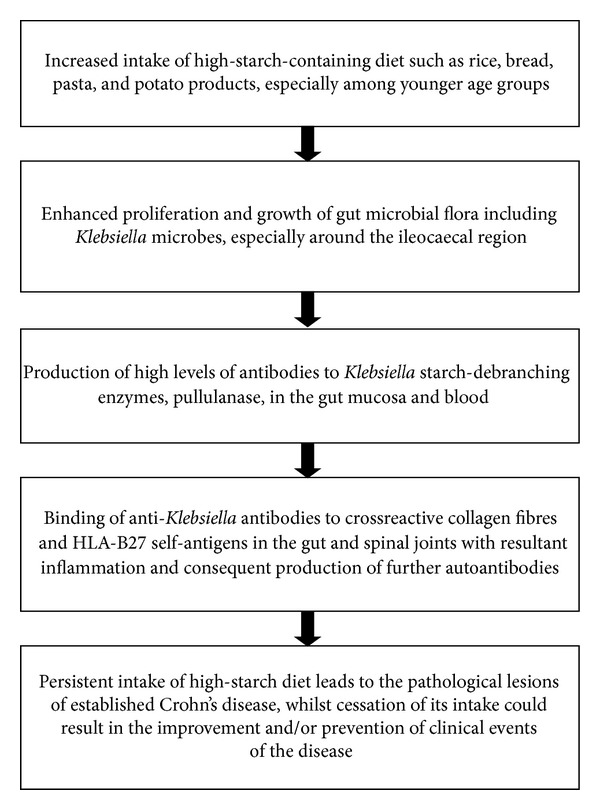
Proposed pathogenetic sequence with the role of *Klebsiella* and starch consumption in the development of Crohn's disease.

A considerable amount of microbiological, molecular, and immunological evidence exists which supports the role of *Klebsiella *microbes in the aetiology of CD (Figure 1) (as shown in the first three lists).

#### 2.2.1. Microbiological and Molecular Studies

- It was reported that, in more than 20% of patients with CD, *Klebsiella* microorganisms have been isolated from the large bowel specimens (Höring et al., 1991) [35]. The disease relapses in patients with CD were found also to be associated with attacks of *Klebsiella oxytoca* colitis [36]. In a study on the biopsy specimens taken from 12 patients with CD, it has been shown that invasion of the bowel mucosa by microbial agents including also Enterobacteriaceae spp. was evident in 55.6% of the ileal and in 25% of the colonic specimens from the CD patients, but no bacteria were detected in the tissues of the non-CD controls [37]. In another study using immunohistochemistry, however, the majority of biopsy specimens taken from the bowel mucosa of patients with IBD have shown negative staining for *Escherichia, Listeria,* and even *Klebsiella* microbes [38]. This latter finding could indicate that it is the microbial bulk in intestinal lumen rather than at the sites of the pathological lesions, which is important in evoking both local mucosal and general antibacterial immune responses.
- *Klebsiella* microbes were found to share molecular similarities with certain self-antigens. For example, nitrogenase reductase enzyme possesses a hexameric amino acid sequence, QTDRED, which has been found to be homologous to another sequence present in HLA-B27 molecules [39]. In a later study, PulD secretion protein from *Klebsiella* pullulanase enzyme, which carries tetramer amino acid residues, DRDE, was found to be homologous to DRED sequence present in HLA-B27 molecules, whilst repeating Gly-X-Pro amino acid sequences present in the pulA secretion components of *Klebsiella* pullulanase enzyme were found to be similar to those present in the trihelical structures of collagen types I, III, and IV [40].
- Significant cytotoxic reactions shown as percentage hemolysis for sheep red blood cells coated with HLA-B27∗05 peptide were detected in the serum samples containing high anti-*Klebsiella* antibodies concentrations taken from AS patients when compared to control sera [41].

#### 2.2.2. Serological and Immunological Studies

Collagen-induced enterocolitis [42] as well as arthritis [43] has been observed in experimental animals when immunized with homologous colonic extracts and collagen type II together with *Klebsiella* lipopolysaccharides.

Elevated levels of humoral immune responses to *Klebsiella* microbes in patients with CD as well as UC have been shown in several studies carried out at six different gastroenterology centres in the United Kingdom.

- Significantly increased levels of antibodies against *Klebsiella* and *Yersinia* enterobacterial agents were observed in patients with CD and UC from Birmingham when compared to corresponding healthy controls [44].
- IgA antibody levels against *Klebsiella *microbes were found to be significantly increased in patients with IBD and AS from Glasgow when compared to corresponding control subjects [45].
- Anti-*Klebsiella* antibody levels were found to be significantly higher in patients with CD and AS from Edinburgh when compared to corresponding controls [46].
- In three consecutive studies carried out at different gastroenterology centres in London and Winchester, similar results have been reported. In one study, *Klebsiella* antibody levels were found to be significantly higher in patients with IBD and AS when compared to healthy or other disease controls, whilst no such elevations were observed in the antibody levels against *E. coli* or other anaerobic intestinal microbes [47]. In a second study, the levels of class-specific isotypic antibodies against many *Klebsiella *antigens were found to be significantly elevated in patients with CD and AS when compared to patients with coeliac disease or to healthy controls [48]. In a third study, the levels of class-specific antibodies against *Klebsiella* microbes and crossreactive collagens I, III, IV, and V were found to be significantly increased in patients with early and late CD as well as in patients with AS when compared to healthy controls. Moreover, a positive correlation was demonstrated between antibody levels to collagen types I, III, and IV and *Klebsiella *in both groups of patients with CD and AS [49].

It is, therefore, plausible that CD disease could result from repeated subclinical infections by *Klebsiella* microbes in the large bowel with the consequent inflammations and tissue damage in the bowel and joints resulting from the binding of anti-*Klebsiella* and anti-self tissue antibodies to the crossreactive targeted antigens.

## 3. Starch Digestion and Its Relation to Gut Microbes

Dietary macronutrients especially carbohydrates are considered as a major factor driving the composition and metabolism of the colonic microbiota. Recent evidence from molecular ecology has shown that the amount and type of nondigestible carbohydrates including starch influence the species composition of the gut microbiota both in short-term dietary intervention and in response to habitual long-term dietary intake [50, 51]. In a recent study, a measure of 120 g/L of glucose was found to be one of the optimal requirements for the cell growth and lipid synthesis of *Lipomyces starkeyi* yeast [52].

Starch is the major dietary carbohydrate for humans. It is composed of two types of structurally distinct *α*-glucan polymer, amylase, and amylopectin. Amylose, which constitutes around 20–30% of the starch particle, consists of long chains of several thousand *α*-glucosyl units joined by *α*(1→4)-linkages, whilst amylopectin, which constitutes the other 70–80% part of starch, is a branched molecule comprising short chains of *α*(1→4)-linked *α*-glucosyl units, joined by *α*(1→6) branch linkages [53]. 

Degradation of the starch molecules is carried out by various catalytic enzymes, which are produced by plants, microbes, and human body. Among these starch-debranching enzymes, however, pullulanases and *α*-amylases [54] are known to be among the potent groups.

*Klebsiella *microbes can survive in harsh environments exploiting some of their enzymatic products, which are required for the protection, maintenance, and survival of these microbes. Apart from nitrogenase reductases, *Klebsiella* can also produce starch-debranching enzymes, such as pullulanases [55]. These microbes can utilize starch as the sole carbon and energy source via two metabolic routes. The first one involves the extracellular degradation into linear maltodextrins by hydrolysis of the glycosidic bonds via the cell-surface-associated pullulanase [56] and then the subsequent cleavage of the glycosidic linkages by the action of the extracellular glycosyltransferase [57]. 

The large bowel in humans is described as a complex ecosystem, containing a large number of microbial species [58]. Although the composition of the colonic microbiota in adults is relatively stable, its concentration, however, can be manipulated by dietary means. For example, it has been reported that a high intake of carbohydrate, particularly oligosaccharides, can stimulate the growth of *Bifidobacterium *spp.*, Klebsiella *spp.*, Clostridium *spp., and *Escherichia coli* in the human colon [59].

Starches are one of the major carbohydrates available in the human colon. A considerable part of the consumed starchy food escapes digestion in the human small intestine and directly enters the large bowel. These starches that are not degraded in the small intestine and referred to as resistant starch can be used as substrate for bacterial fermentation [60]. 

It has been shown that up to 20% of the total starch materials consumed by normal individuals fail the absorption process when assessed by oral hydrogen excretion studies [61]. These starch materials are basically present in potatoes and wheat flour products such as pasta and bread (as shown in the lists below).

*Recommended Diet for Crohn's Disease and Ankylosing Spondylitis Patients*

*Decreased Intake of “High-Starch-Containing Foods”*

- *Flour and related products*: breads, biscuits, cakes, puddings, pies, and popcorn.
- *Pasta products*: macaronis, noodles, spaghetti, pizzas, and pastry.
- *Rice varieties*: brown or white, boiled, fried, or in puddings.
- *Potatoes*: baked, boiled, fried, roasted, or mashed potatoes.

*Increased Intake of “None-Low-Starch-Containing Foods”*

- *Meat*: beef, pork, lamb, bacon, salami, corned beef, luncheon mean, potted meat, ham, and veal as well as chicken, turkey duck, or any other poultry meat.
- *Fish*: white fish such as cod, haddock, plaice, and sole; shellfish such as crab, lobster, prawns, scampi, cockles, mussels, and oysters; and other fish such as herring, salmon, mackerel, tuna, and sardines.
- *Milk products*: fresh, dried, or condensed milk, plain and flavoured yoghurts, and all types of cheese.
- *Eggs*: prepared in any manner.
- *Vegetables*: green vegetables such as cabbages, cauliflowers, sprouts, courgettes, peppers, mushrooms, carrots, and spinach or all salad vegetables like lettuce, cucumber, tomatoes, and water cress.
- *Fruits*: any types of fruits.

The total colony counts in the bacterial population of bifidobacteria, lactobacilli, streptococci, and enterbacteria species were found to be increased significantly in the caecum and faeces of a group of rats which have been fed resistant potato starch when compared to those rats taking a diet made of rapidly digestible waxy maize devoid of resistant starch [62]. 

In an in vitro study, it has been found that the mean number of *Klebsiella* was ten times higher when incubated with simple carbohydrate products such as sucrose, lactose, and glucose than with eleven different amino acids [63]. Furthermore, *Klebsiella* microbes do not seem to survive or grow on plant cellulose materials [64]. In a recent experimental study, rats which have been fed potato starch diet had higher colonic bacterial loads than those on cellulose only diet [65].

These results indicate that complex carbohydrates such as starch products are necessary for the growth, replication, and persistence of *Klebsiella* as well as other enterobacterial agents in the gut.

## 4. Proposal for the Use of Antimicrobial Measures in Treatment of CD

Patients with CD usually require lifelong heavy medications and devastating surgeries. The current treatment involving the use of anti-inflammatory, immunosuppressive, and biologic modalities [66] has significant limitations due to lack of treatment response in some patients as well as the occurrence of adverse side effects, such as increased risk of infection [67] and malignancy [68] among some other patients especially in those taking antitumor necrosis factors. Some patients with CD, however, may also need surgical interventions [69]. 

Sustained therapeutic responses in patients with CD, however, may require more radical and comprehensive approaches involving strategies which help in the modification of the intestinal bacterial microenvironments. A solution for this medical predicament is the use of an alternative therapy, which should be harmless and aimed at the elimination of the most plausible microbial agent, such as *Klebsiella*, in order to improve or even halt the inflammatory and pathological damage occurring in patients with CD. 

To achieve this therapeutic goal, the most likely strategy which could be added to the management of this disease is the use of antimicrobial agents and dietary manipulation.

### 4.1. Antimicrobial Measures

Manipulation of luminal content of the gut using antibiotics may represent a potentially effective therapeutic option. Whilst antimicrobials were shown to be clinically effective mainly in preventing postoperative recurrence [70], there could also be potential for their inclusion in the primary treatment of CD [71].

In a review analysis, the trial of an antibacterial agent, rifaximin, has been shown to provide promising results in inducing remission of CD in up to 69% in open studies and significantly higher rates than placebo in double blind trials [72]. In another meta-analysis review of randomized placebo-controlled studies, it has been shown that there was a statistically significant effect of antibiotics being superior to placebo in patients with active and quiescent CD as well as active UC [73]. Furthermore, the results of another study have shown that administration of 800 mg of the extended intestinal release form of rifaximin twice daily for 12 weeks has induced remission in patients with moderately active CD [74]. 

It is possible that the use of *Klebsiella* sensitive antibiotics could reduce the bacterial loads in the bowel of CD patients and prevent production of further anti-*Klebsiella* antibodies and tissue damaging autoantibodies.

### 4.2. Low-Starch Diet

Although no studies have yet been carried out on patients with CD in regard to the use of low-starch diet, clinical trials, however, have been carried out in AS patients with some encouraging results. In a longitudinal clinical study carried out on a group of patients with AS receiving low-starch diet, there was a significant drop in the erythrocyte sedimentation rate and total serum IgA by the end of the 9-month period of the study. Most of these patients have reported positive responses to the diet involving partial to complete recovery, ranging from disappearance of symptoms to a drop or even cessation in the intake of anti-inflammatory drugs [75]. From experience of recommending the low-starch diet in patients with AS for the last 3 decades and more recently in CD, it is concluded that normally it takes around six to eight months for the diet to show its effects [76].

It is plausible that a drop of the starch intake in the daily dietary consumption might reduce the bowel microflora by depleting the substrates necessary for enterobacterial growth. For example, reducing starch intake could stop the growth of *Klebsiella*, with a possibility of having beneficial effects on the disease outcome.

## 5. Conclusion

It is concluded that *Klebsiella* has a pivotal role in the aetiopathogenesis of CD, as evidenced by enhanced anti-*Klebsiella* immune responses and significant cross-reactivity and cytotoxic reactions. Low-starch diet could help in the eradication of *Klebsiella* in the bowel and, hence, decreasing the disease activity and progress with eventual halt or regression of the pathological process in patients with CD and AS.
